# Weekly variation in markers of cardiometabolic health – the possible effect of weekend behavior – a cross-sectional study

**DOI:** 10.1186/s12872-020-01692-x

**Published:** 2020-09-07

**Authors:** Louise Sølund Hansen, Marlene Rosager Lund Pedersen, Jakob Tarp, Anna Bugge, Niels Wedderkopp, Niels Christian Møller

**Affiliations:** 1 Department of Sports Science and Clinical Biomechanics, University of Southern Denmark, Campusvej 55, 5230 Odense M, Denmark; 2 Department of Sports Medicine, Norwegian School of Sports Sciences, Oslo, Norway; 3 Department of Midwifery, Physiotherapy, Occupational Therapy and Psychomotor Therapy Faculty of Health, University College Copenhagen, Copenhagen, Denmark; 4 Orthopedic dep. Hospital of South West Denmark, Department of Regional Health Research, University of Southern Denmark, Esbjerg, Denmark

**Keywords:** Cardiometabolic risk factors, Weekday differences, Healthy adolescents, Health-related behavior, Physical activity, Diet

## Abstract

**Background:**

Adolescents’ health-related behavior varies from weekday to weekend. Only few studies, however, have examined to which degree such variation will affect markers of cardiometabolic health. Therefore, the primary aim of this study is to examine if markers of cardiometabolic health differ between different days of the week in adolescents.

**Methods:**

This cross-sectional school-based study included up to 581 participants, 11–17 years old. Markers of metabolic health were insulin, glucose, triglyceride, HDL-cholesterol (HDL-C), LDL-cholesterol (LDL-C) and blood pressure. Linear mixed regression modelling was used to examine the cardiometabolic profile across weekdays.

**Results:**

Significant declining trends were observed across the week in adolescents’ levels of cardiometabolic health markers. Lower levels of insulin (16.1%), glucose (2.6%) and triglyceride (24.7%) were observed on Fridays compared to Mondays (*p* ≤ 0.006). Gradual improvement in measurement profiles across weekdays was less apparent for HDL-C, LDL-C, systolic blood pressure and diastolic blood pressure (*P* ≥ 0.06). Analyses stratified by sex suggested a more noticeable pattern of gradual improvement across weekdays in boys than in girls.

**Conclusion:**

Significantly lower levels of insulin, glucose and triglyceride were observed in adolescents on Fridays compared to Mondays. However, when sex specific analyses were performed significant profile variations were only observed across the week in boys. More research is needed to better understand which behavioral factors in particular seem to influence weekly variation in markers of cardiometabolic health - especially since such variation potentially will have an impact on how assessments of markers of cardiometabolic health optimally should be planned, standardized and carried out, both in research and in medical practice.

## Background

Being physically active is important for maintaining good cardiometabolic health [1, 2] and for prevention of many non-communicable diseases and overall mortality [3]. Exercise intervention studies have shown favorable changes in HDL-C, triglycerides, insulin levels and waist circumference in obese children [4] as well as lower systolic blood pressure in adolescents with no health problem or clinical diagnose [5]. A large cross-sectional study, including pooled data from 14 studies and 20.871 children and adolescents found favorable levels of waist circumference, systolic blood pressure, insulin, and HDL-C among children and adolescents who spent more time in moderate to vigorous physical activity (MVPA), independent of the amount of time spent sedentary [6]. The evidence from prospective studies also supports a protective association between physical activity (PA), overall sedentary time and cardiometabolic risk-markers in children and youth [7–9]. Other modifiable behavioral factors influencing markers of cardiometabolic health during childhood and adolescence include sleep-duration and energy intake [10–15].

Studies have shown that children’s health related behavior varies from weekday to weekend. At weekends, children tend to be more sedentary, sleep less, be less physically active, consume more sugar-sweetened beverages and have higher total energy intake when compared to weekdays [16–19].

A Danish study [20] has shown that markers of metabolic health were about 30% higher in 8–11-year-old children when measured on Mondays as compared with measurements taken on Fridays. Hjorth and colleagues hypothesized that this variation was explained by lower PA and sleep duration during the weekend, resulting in elevated levels of the conventional markers of metabolic health. A gradual improvement in the metabolic profile (insulin resistance, triglycerides, and leptin) was observed over the week. The same pattern was observed for fasting triglyceride levels among 9–26-year-old in- and out-patients in the Capital region of Denmark [21]. However, more research in other samples including a wider array of outcomes is needed to confirm if measurements of children’s and young people’s health show systematic variation across weekdays. Adolescence is characterized by growing independence and less parental control as compared with childhood, which is likely to influence variation in various weekday-weekend behaviors. Therefore, an examination of potential weekday variation in markers of cardiometabolic health in adolescents is warranted.

Systematic variation in results from blood samples drawn at different days of the week could impact the validity of data being analyzed in cohort studies or experimental interventions. Same consequences might also apply to hospitals and medical centers. Systematic variations in blood profiles across different weekdays could potentially lead to adverse consequences and health effects in individuals undergoing screening of their health status based on the results of markers of metabolic health observed in the blood. If substantial systematic variations in blood profiles across weekdays are confirmed in multiple samples, it dictates a need of more standardized blood sampling protocols, or alternatively that results from blood samples are being scaled to specific days of reference. It could, therefore, influence the procedure for when to obtain blood samples during the week or how to handle statistical analyses e.g., in research projects.

The aim of this study is to examine if adolescents’ markers of cardiometabolic health differ across different days of the week. We hypothesize that the least favorable health profile is observed in adolescents examined in the beginning of the week then followed by a gradual improvement observed in individuals examined Tuesdays to Fridays.

## Methods

### Study design and participants

This cross-sectional study was nested in the Childhood Health, Activity and Motor Performance School Study in Denmark (CHAMPS-study) which is an ongoing quasi-experimental trial, evaluating the effects of augmented physical education in public school students. The study began in 2008 and included 10 public schools. The CHAMPS-study sample and procedures have been described in detail previously [22].

This study uses data from CHAMPS III, the 2015 follow-up of the original cohort. A total of 1452 eligible subjects, aged 11–17 years, were invited in CHAMPS III and 745 participants provided written informed consent from a parent or legal guardian. Measurements were taken from February to May 2015 and were performed at the schools by trained staff following standardized protocols. Participants were mainly examined with their classmates, i.e. participants from one class on Mondays, a new class on Tuesdays, etc. The test order was based on convenience. Rescheduling was sought for participants who were unavailable on their class test-day.

The study was approved by the ethnical committee, Region of Southern Denmark (ID S-20080047) and registered at the Danish Data Protection Agency (J.nr. 2008-41-2240).

### Measurements

#### Blood samples

Fasting blood samples were obtained between 8.00 AM and 10.00 AM. Before drawing the blood samples, participants were offered a local anesthetic. Trained phlebotomists handled all blood samples. The samples were kept on ice and handed to the laboratory within 4 h. The samples were kept at − 80 °C until analyzed for insulin, glucose, triglyceride and cholesterol. Total cholesterol, triglyceride, glucose and HDL-C were analyzed by quantitative determination using enzymatic, colorimetric method on Roche/Hitachi cobas c system (Roche, Mannheim, Germany). Insulin was analyzed using solid phase enzyme labeled chemiluminescent immunometric assay. LDL-C was calculated using the Friedewald equations [23]. These analyzes were conducted at the Centre for Sport Science and University Sports, University of Vienna.

Not all adolescents had blood samples drawn on their class test-day due to non-fasting, in which case rescheduling of the blood sample was sought. Participants with no fasting blood sample were excluded from analysis of blood-based health markers.

#### Blood pressure

Blood pressure was measured with a suitable cuff size on the left arm using an automized blood pressure monitor (OMRON705IT). Before the blood pressure was measured, the adolescents rested for 5 min in a sitting position. Measurements were performed five time with two-minutes intervals while the subject was seated. If the pressure continued to fluctuate more recordings was required until the difference between two subsequent recordings was < 6 mmHg. The mean of the last three values of systolic and diastolic blood pressure was used in the analysis.

#### Anthropometry

Body weight was measured to the nearest 0.1 kg on an electronic scale (Tanita BWB-800, Tokyo, Japan). Adolescents were wearing shorts, t-shirts and no shoes.

Height was measured to the nearest 0.5 cm using a stadiometer (Harpenden stadiometer, West Sussex, UK). Adolescents were barefooted. The mean of the two measurements was used in the analysis.

Body Mass Index (BMI) was calculated as weight (kg)/height (m)^2^.

#### Sexual maturity

Pubertal status (Tanner stage) was classified into five different categories according to the methods described by Marshall and Tanner [24, 25], and was self-reported by the adolescents (Additional file 1). Less than 1% reported Tanner stage 1, therefore in this study Tanner stage was combined into two groups; pre- or middle-pubertal (Tanner stage 1–3) and pubertal (Tanner stage 4–5). Breast development and pubic hair status were used for girls and boys, respectively.

#### Aerobic fitness

Aerobic fitness was assessed using the Andersen-test. Participants were instructed to run as fast as they could between two parallel lines 20 m apart, touch the floor behind the line with one hand, turn around and run back again. The test consisted of 15 s of running followed by 15 s of rest for a total of 10 min. The total distance (meters) covered by the adolescents was used as a proxy for their maximal aerobic performance [26].

#### Socioeconomic status

The parents reported their highest educational level in a questionnaire (Additional file 2). We used the mothers’ education as an indicator of socioeconomic status (SES) [27]. The level of education was divided into two categories, non-tertiary education and tertiary education. Non-tertiary education included lower secondary (≤10 years), upper secondary (12 years) and vocational education (12 years). Tertiary education included short higher (14 years), medium higher (15–16 years) and long higher education (≥17 years).

### Data analysis and statistics

We used a linear mixed regression model to examine differences in markers of metabolic health in adolescents measured at different weekdays. Potential overall differences in profiles of insulin, glucose, triglyceride, cholesterol and blood pressure across the weekdays were tested using a Wald test. If this test found evidence against identical values on all days, we post hoc tested whether mean values obtained on Tuesdays to Fridays differed from values obtained on Mondays. We also tested for linear trends across all weekdays. Two samples were analyzed: one based on availability of fasting blood health-markers and one based on availability of blood pressure. This approach was chosen because some participants preferred not to provide a blood sample.

Model assumptions of normal distribution of residuals and variance homoscedacity were examined and met. All analyzes were adjusted for age, gender, BMI, Tanner stage, aerobic fitness, SES, and type of school (intervention- or control school) because the schools were tested in a practical, not randomized, order. We included school classes as random effects. All analyzes were repeated with stratification by sex. The level of significance was set at *P* ≤ 0.05. All statistical analyzes were conducted using STATA 14.0.

## Results

In total 581 participants were available after exclusion of subjects based on missing variables - 497 participants with valid blood samples and 581 participants with valid blood pressure assessments.

Baseline characteristics are presented in Table 1. The percentage of boys included in the study was 50.1% and the mean (SD) age was 14.1 (1.2).

**Table 1:** Baseline characteristics

	Boys (*n* = 294)	Girls (*n* = 287)
Age (years)	14.3 (1.2)	14.0 (1.2)
Height (cm)	169.0 (11.2)	164.2 (7.1)
Weight (kg)	55.5 (12.2)	53.3 (9.3)
BMI (kg/m^2^)	19.2 (2.5)	19.7 (2.7)
**Tanner stage (%)**		
Stage 1–3	40	46
Stage 4–5	60	54
Insulin (μlU/mL)^a^	5.6 (4.1–7.6)	7.0 (5.4–9.1)
Glucose (mg/dL)	89.0 (6.3)	88.4 (7.8)
Triglyceride (mg/dL)	63.9 (26.0)	70.2 (28.4)
HDL-C (mg/dL)	53.9 (13.1)	57.6 (13.2)
LDL-C (mg/dL)	83.6 (21.6)	91.7 (23.0)
Systolic BP (mmHg)	109.2 (9.1)	105.6 (8.0)
Diastolic BP (mmHg)	61.9 (6.2)	63.5 (6.0)
**Socioeconomic status (%)**		
No tertiary qualifications	35	31
Tertiary qualifications	65	69

*N* = 581 except for biochemical biomarkers (*n* = 497). Values are mean (SD) unless otherwise noted

^a^median (25th and 75th percentiles)

Tables 2 and 3 show how drawings of participants’ blood samples and assessments of their blood pressure were distributed across the different weekdays (i.e., Monday to Friday). Collection of blood samples and blood pressure data were distributed unequally across weekdays with fewest assessment performed on Mondays (blood sample: 12.2% of total sample, blood pressure: 14.8% of total sample) and most on Fridays (blood sample: 23.9% of total sample, blood pressure: 23.4% of total sample). The distribution of gender, age, BMI, sexual maturity and SES did not differ across weekdays (*P*-values ≥0.34). Participants performed slightly better in the Andersen-test on Wednesday and Friday as compared with Mondays (*P* ≤ 0.05).

**Table 2:** Distribution of variables (Monday to Friday) for blood samples

	Weekday of blood samples (*n* = 497)	Boys (%)	Age (years)	BMI	Andersen-test (m)
Monday	61 (12.2%)	46	14.2 (1.3)	19.5 (2.3)	1050 (114)
Tuesday	100 (20.1%)	48	13.9 (1.1)	19.3 (2.5)	1080 (101)
Wednesday	115 (23.1%)	53	14.2 (1.2)	19.3 (2.5)	1104 (106)*
Thursday	102 (20.5%)	53	14.2 (1.3)	19.4 (2.7)	1098 (113)
Friday	119 (23.9%)	54	14.4 (1.0)	19.7 (2.8)	1109 (116)*

Mean (SD). *denotes significant difference from Monday (*P* ≤ 0.05)

**Table 3:** Distribution of variables (Monday to Friday) for blood pressure

	Weekday of blood pressure (*n* = 581)	Boys (%)	Age (years)	BMI	Andersen-test (m)
Monday	86 (14.8%)	50	14.2 (1.3)	19.4 (2.7)	1072 (114)
Tuesday	115 (19.8%)	46	13.8 (1.1)	19.1 (2.4)	1069 (99)
Wednesday	122 (21.0%)	52	14.1 (1.3)	19.4 (2.3)	1107 (103)*
Thursday	122 (21.0%)	52	14.1 (1.3)	19.4 (2.7)	1089 (110)
Friday	136 (23.4%)	52	14.4 (1.0)	19.7 (2.9)	1105 (121)*

Mean (SD). *denotes significant difference from Monday (*P* ≤ 0.05)

Evidence of weekday variation was observed for insulin (*p* = 0.006), glucose (*p* = 0.003), and triglyceride (*p* < 0.001). No evidence of weekday variation was observed for HDL-C (*p* = 0.09), LDL-C (*p* = 0.467), systolic blood pressure (*p* = 0.113) and diastolic blood pressure (*p* = 0.446). Insulin, glucose and triglyceride levels were 16.1, 2.6 and 24.7% lower on Fridays compared to Monday, respectively (*P* ≤ 0.04). The differences between Mondays and Fridays for HDL-C (3.3% higher), LDL-C (6.8% higher), systolic blood pressure (1.8% lower) and diastolic blood pressure (0.03% lower) did not reach statistical significance (*P* ≥ 0.06). Values for markers of cardiometabolic health are presented in Table 4. When we repeated our analyses with weekday entered as a continuous variable, levels of insulin, glucose, and triglycerides showed evidence of a linear decreasing trend from Monday to Friday (*p* < 0.01) but this was not the case for HDL-C (*p* = 0.06), LDL-C, systolic BP and diastolic BP (*p* > 0.11).

**Table 4:** Values of metabolic health markers across weekdays

	Monday	Tuesday	Wednesday	Thursday	Friday	P-value
**Insulin (**μlU**/ml)**	7.5 (6.6, 8.3)	7.7 (7.1, 8.4)	6.6 (6.1, 7.2)	6.4 (5.8, 7.0)	6.3 (5.7, 6.8)	0.006
Difference from Monday, absolute (%)		0.3 (3.8%)	−0.8 (−11.0%)	−1.1 (− 14.3%)*	−1.2 (− 16.1%)*	
**Glucose (mg/dl)**	89.5 (87.7, 91.3)	90.6 (89.2, 92.0)	87.7 (86.4, 88.9)	89.3 (88.0,90.7)	87.2 (85.9,88.4)	0.003
Difference from Monday, absolute (%)		1.1 (1.2%)	−1.8 (−2.0%)	− 0.1 (− 0.1%)	− 2.3 (− 2.6%)*	
**Triglyceride (mg/dl)**	80.7 (73.8,87.6)	67.1 (61.8, 72.5)	69.9 (65.0, 74.7)	62.5 (57.2, 67.8)	60.7 (55.9, 65.6)	< 0.001
Difference from Monday, absolute (%)		−13.5 (− 16.8%)*	−10.8 (− 13.4%)*	−18.2 (−22.5%)*	−20.0 (− 24.7%)*	
**HDL-C (mg/dl)**	54.9 (51.7,58.1)	54.2 (51.7, 56.6)	54.3 (52.1, 56.6)	58.3 (55.9, 60.8)	56.7 (54.5, 58.9)	0.09
Difference from Monday, absolute (%)		−0.7 (−1.3%)	− 0.6 (− 1.0%)	3.4 (6.3%)	1.8 (3.3%)	
**LDL-C (mg/dl)**	84.0 (78.3,89.6)	86.3 (81.9, 90.6)	90.1 (86.1, 94.0)	86.9 (82.6, 91.2)	88.2 (84.3, 92.2)	0.34
Difference from Monday, absolute (%)		4.2 (5.6%)	6.8 (8.9%)	4.9 (6.4%)	6.8 (8.9%)	
**Systolic BP (mmHg)**	109.3 (107.6110.9)	106.3 (104.9, 107.7)	107.6 (106.2, 108.9)	107.1 (105.8, 108.5)	107.3 (106.0, 108.6)	0.11
Difference from Monday, absolute (%)		−2.9 (−2.7)*	−1.7 (−1.6%)	−2.1 (−1.9%)*	−1.9 (− 1.8%)*	
**Diastolic BP (mmHg)**	63.1 (61.6,64.5)	63.3 (62.1, 64.6)	62.7 (61.5, 63.8)	61.7 (60.6, 62.9)	60.9 (61.7, 64.0)	0.45
Difference from Monday, absolute (%)		0.3 (0.4%)	−0.4 (−0.7%)	−1.3 (−2.1%)	−0.2 (− 0.3%)	

*P*-value: Global differences between the weekdays using a Wald test

Data are predictive margins (95% CIs) with adjustment for age, gender, BMI, Tanner stage, aerobic fitness, socioeconomic status, type of school (intervention or control) and class cluster. *denotes significant difference from Monday as well as overall significant declining trend from Monday to Friday (P ≤ 0.05)

**Fig. 1:**
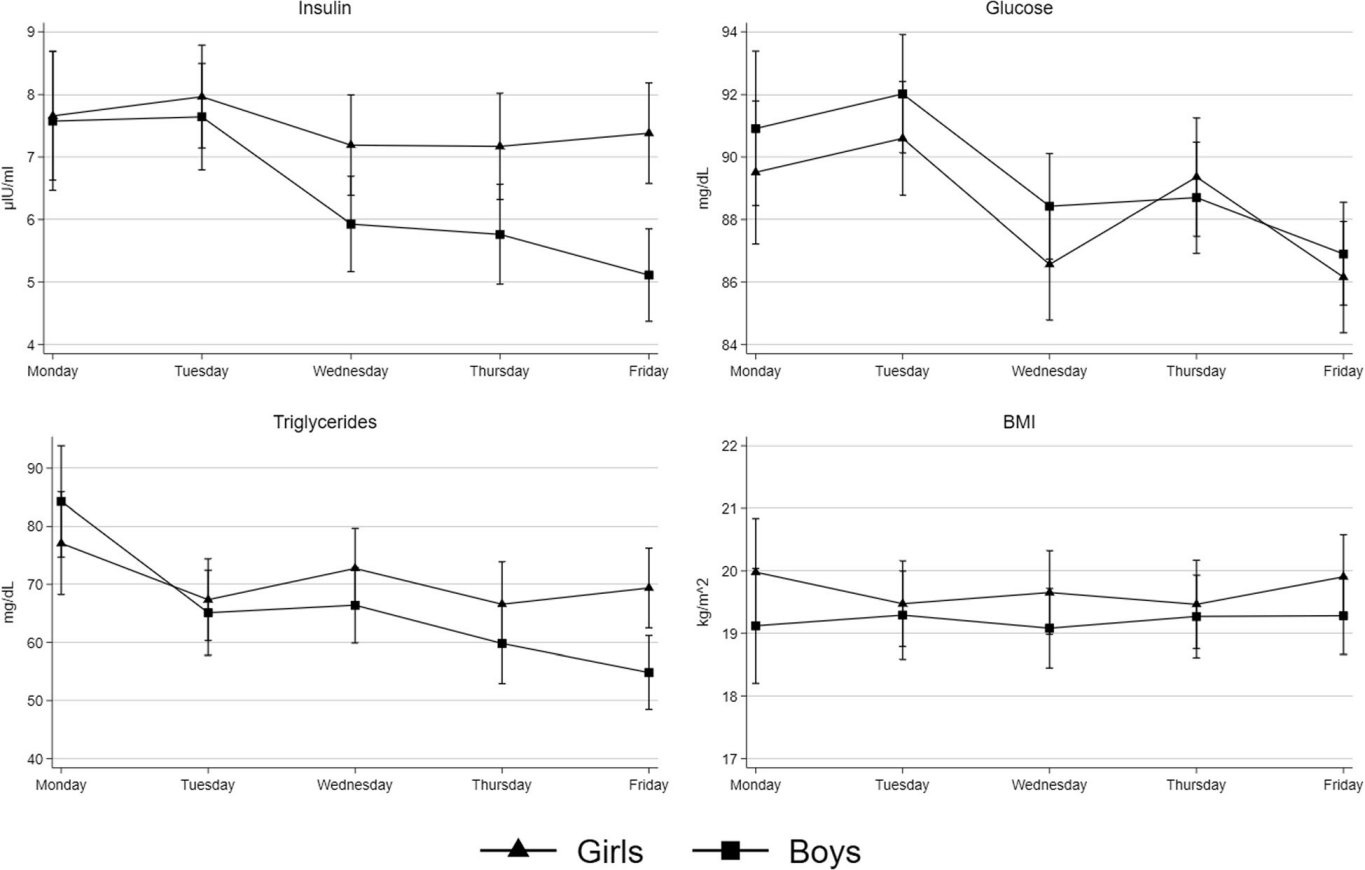
Sex-stratified levels of insulin, glucose, triglycerides and BMI across weekdays in Danish adolescents. Values are predictive margins (95% CIs) with adjustment for age, sex, BMI, Tanner stage, aerobic fitness, socioeconomic status, type of school (intervention or control) and class cluster

When stratified by sex, variations across the weekdays were less pronounced in girls than in boys, particularly for insulin. Levels of insulin, glucose triglyceride, and BMI (as a negative control outcome) observed across the different weekdays in boys and girls are shown in Fig. 1.

## Discussion

A significant declining trend was found in levels of insulin, glucose, and triglyceride in adolescents throughout the week with the lowest levels being observed on Fridays. Stratifying by sex suggested that the gradual improvement in cardiometabolic health-profile across the week was more evident in boys than in girls.

In a study by Hjorth et al. [20], where levels of HOMA-IR and triglyceride were compared across different days of the week in 8 to 11-year-old children, results showed a decline of 35 and 28%, respectively, from Mondays to Fridays. In our study, we extend these observations to the adolescent age-group. Compared to the results observed by Hjorth and colleagues, we observed a less decline in insulin levels between Monday and Friday (16% vs 35%) but a similar decline in triglycerides levels (25% vs 28%). These differences in weekly variations in insulin profiles might be explained by our participants being older than the participants included in the study by Hjorth and colleagues, or by differences in PA levels or dietary patterns.

Based on the results observed in 1.8 million blood samples, Jaskolowski and colleagues [21] reported that triglyceride levels were highest immediately after the weekend with gradually lower levels being observed during the following weekdays. The samples were commissioned by general practitioners, specialists and doctors during everyday life or hospitalization. Largest variations were observed in out-patients between the age of 9 to 26 years with up to 20% higher values being observed on Mondays when compared to Fridays, which is similar to the results observed in our study. No significant fluctuation was observed in total-cholesterol, LDL-C and HDL-C, which is consistent with our results.

A systematic variation in the cardiometabolic health-profile in children and adolescents across the week could have profound implications for how best to design research projects and subsequent analyze collected data. Variation in measured triglycerides from 80.7 mg/dl observed on Mondays to 60.7 mg/dl observed on Fridays suggests that measurement error will be reduced substantially by standardizing the days in which blood samples are drawn. This could be done by either ensuring that assessments of individuals are performed on the same weekday or by excluding the first days of the week as viable options for collecting data. This would obviously decrease flexibility in research projects. If weekend-behaviors exhibit a potent negative influence on measured values (i.e. up to 25%), this stimulus may potentially be strong enough to override the influences of interventions with smaller stimuli, e.g. the effects of school-based interventions were typically only minor changes to the curriculum are made. Furthermore, these variations may have implications for health care, and therefore for public health, if blood samples taken on different days are not comparable and absolute cut-offs are used to determine disease risk or status.

We observed no systematic variations in HDL-C, LDL-C and blood pressure profiles across different days of the week. These markers of cardiometabolic health may not be fluctuating transiently to the same extend as insulin, glucose or triglyceride. A study by Altenburg et al. [28] reported that breaking up prolonged sedentary time with 8 min of cycling every hour for 8 h did not alter HDL-C, LDL-C and total cholesterol levels in young adults. This might indicate that even if the adolescents are more sedentary in the weekends compared to weekdays, it might not be enough to influence cholesterol levels.

Some studies find that high amounts of MVPA influence levels of cholesterol and blood pressure [5, 29, 30] indicating that a certain intensity, frequency and/or duration of PA is needed, before cholesterol and blood pressure profiles are influenced. In the study by Taylor-Tolbert et al. the acute response of one training session on blood pressure was investigated. They observed an acute blood pressure response 24 h post exercise in older hypertensive men [30]. This does not support the results observed in our study, which did not indicate any systematic weekly variation in the blood pressure profile. The difference between results observed in in our study and results observed in the study by Taylor-Tolbert and colleagues could be due to that participants in the latter being older and all diagnosed with hypertension. Furthermore, it should be noticed that results observed in the study by Tayloy-Tolbert et al. represents the effect of a more structured training regime carried out, and supervised, in the laboratory at a certain intensity (approx. 70%) relative to the individuals’ VO2max.

In our study, insulin, glucose, and triglyceride levels seem more volatile, and measurement profiles could potentially be influenced if the adolescents’ behavior varies between weekdays and weekends [16, 17, 19]. Studies have shown that even 2 min of breaking up prolonged sedentary time every 20 min in adults [31], or 1 min and 40 s of sedentary time substituted with low-moderate training every 15 min in young adults [32], will lower the levels of insulin and glucose postprandial. However, triglyceride levels did not differ significantly as a result of subjects participating in training in the study by Peddie and colleagues [32]. This is in contrasts to the results observed in the present study as we saw the largest decrease (24.7%) from Monday to Friday in levels of triglyceride. An acute response of endurance training on triglyceride levels has previously been demonstrated in young individuals and adults. In blood samples which were obtained 24 h following the last exercise session, triglyceride levels were observed to be lower than at baseline, but no changes were observed in blood samples obtained 72 h post training [29]. This indicates that triglyceride is volatile and is acutely influenced by endurance training. However, these findings primarily applied for adults and any comparison with our results should therefore be performed with caution.

Studies have shown that MVPA have an acute effect on levels of insulin and glucose. In a previous study by King et al. [33] the acute response of seven-days interruption to training on glucose tolerance and insulin action was assessed in moderately trained middle-aged individuals. During 5 days before testing, participants performed 45 min daily exercise at about 70% VO2max. In the following 7 days, participants were inactive and were tested on these 7 days. The study showed that improved insulin sensitivity persisted for 3 days after the last training session, but not for five or 7 days, suggesting that the frequency of exercise needed to maintain the exercise-induced improvement in glucose tolerance is, at least, once every third day. Boule et al. [34] confirmed these findings in more than 500 previously sedentary men and women by reporting lower fasting insulin compared to pretraining 24 h after the last exercise bout in a 20-week training program. This improvement, though, was no longer evident 72 h after the last bout of training.

Our results suggest that variations throughout the week in markers of cardiometabolic health are not identical in adolescent boys and girls. This might indicate greater variation throughout the week in health-related behavior in boys than in girls. Boys are more physically active than girls [35], and both girls and boys are more physically active on weekdays compared to weekends [16]. In addition, boys have a significantly higher total energy intake than girls during the week [19]. Similar findings have recently been confirmed in a nationwide Danish study where lower PA and dietary quality was reported in the weekends compared to the weekdays. The weekly variation appeared large especially among the younger individuals, whereas gender differences were modest [18]. Interesting, though, other studies previously conducted in Danish children have indicated that gender differences in PA levels might be more noticeable during the weekdays as compared to the weekends [36, 37]. Also, a recent study conducted in Finish adolescents indicates a more marked increase in sedentary behavior in boys compared to girls during adolescence, especially during the weekend days [38]. Therefore, we hypothesize, that the differences which were observed between boys and girls in our study regarding variations in markers of cardiometabolic health might be due to sex-specific patterns in health-related behaviors especially evident at certain days of the weeks.

Strengths in our study include a large and well-characterized sample of adolescents with blood samples and blood pressure obtained following a standardized protocol. Furthermore, other data collected simultaneously (i.e., both demographic and socio-demographic information) allowed us to adjust for several relevant covariates thereby increasing comparability among individuals when examining markers of cardiometabolic health across different days of the week.

This study also has some limitations. Participants were measured at different days of the week, and the assumption is that there are no overall systematic differences between the participants that were measured at the different days. The plausibility of this assumption is supported by the fact that no differences in age, sex, and BMI was being observed across weekdays. However, some variation in cardiorespiratory fitness was observed with participants measured on Fridays performing about 6% better than their peers measured on Mondays. We speculate if this variation was random - importantly though, this variation was controlled for in all analyses.

Although we used BMI as a negative control outcome in our study, the inclusion of a control group where less variation in health-related behavior is expected would have strengthen study quality by adding another dimension of negative control to the observed results. Therefore, for future research we recommend including data collected in adolescents e.g., during summer vacation where subjects’ weekday and weekend days behaviors might differ less compared to their behaviors during the school year. For future research, it will also be relevant to obtain more detailed and repeated information on markers of cardiometabolic health and health-related behaviors assessed in the same subjects throughout the complete week in large and diverse groups of participants in order to validate the amount of weekly behavioral variation and the strength of association with health at the individual level.

## Conclusions

In this study a significant declining trend was observed across the week in adolescents’ levels of insulin, glucose and triglyceride with the lowest levels being observed on Fridays compared to Mondays. When stratifying by sex the gradual improvement in health markers appeared more evident in boys than in girls. Potentially, these findings could have substantial clinical implications – e.g., when subjects are being screened for need of treatment or medication in medical centers, or when research data are being analyzed and interpreted. More research is needed to: a) systematically examine which health-related behavior in various samples of individuals in particular seem to influence variations in markers of cardiometabolic health across different days of the week, and b) determine to which degree variations in profiles of these health markers will impact daily clinical practice and scientific research conclusions.

## Supplementary information

**Additional file 1 Supplementary file 1.** Self-assessment of pubertal status. Tool used in the CHAMPS study-DK III for judging participants’ sexual maturity

**Additional file 2 Supplementary file 2.** Parents’ education and type of work. Questionnaire applied in the CHAMPS study-DK III for obtaining information on parental socioeconomic position

## Data Availability

The data supporting the conclusions of this article are stored in the Danish National Archives. Data can be available upon request from the CHAMPS-DK steering committee due to legal and ethical restrictions. Interested parties may contact Dr. Niels Wedderkopp (nwedderkopp@health.sdu.dk). The following information is mandatory at the time of application: a description of how data will be used, securely managed and stored, and finally permanently deleted.

## Abbreviations

HDL-C: HDL-cholesterol
LDL-C: LDL-cholesterol
MVPA: Moderate to vigorous physical activity
PA: Physical activity
CHAMPS-study: The childhood health, activity and motor performance school study
BMI: Body mass index
SES: Socioeconomic status

## References

[1] Garber CE, Blissmer B, Deschenes MR, Franklin BA, Lamonte MJ, Lee IM, Nieman DC, Swain DP (2011). American College of Sports M: American College of Sports Medicine position stand. Quantity and quality of exercise for developing and maintaining cardiorespiratory, musculoskeletal, and neuromotor fitness in apparently healthy adults: guidance for prescribing exercise. Med Sci Sports Exerc.

[2] Roberts CK, Hevener AL, Barnard RJ (2013). Metabolic syndrome and insulin resistance: underlying causes and modification by exercise training. Compr Physiol.

[3] Lee IM, Shiroma EJ, Lobelo F, Puska P, Blair SN, Katzmarzyk PT (2012). Lancet physical activity series working G: effect of physical inactivity on major non-communicable diseases worldwide: an analysis of burden of disease and life expectancy. Lancet.

[4] Zorba E, Cengiz T, Karacabey K. Exercise training improves body composition, blood lipid profile and serum insulin levels in obese children. J Sports Med Phys Fitness. 2011;51(4):664–9. 22212270

[5] Buchan DS, Ollis S, Thomas NE, Buchanan N, Cooper SM, Malina RM, Baker JS. Physical activity interventions: effects of duration and intensity. Scand J Med Sci Sports. 2011;21(6):e341–350. 10.1111/j.1600-0838.2011.01303.x 21518010

[6] Ekelund U, Luan J, Sherar LB, Esliger DW, Griew P, Cooper A. Moderate to vigorous physical activity and sedentary time and cardiometabolic risk factors in children and adolescents. Jama. 2012;307(7):704–12. 10.1001/jama.2012.156 PMC3793121 22337681

[7] Tarp J, Brond JC, Andersen LB, Moller NC, Froberg K, Grontved A (2016). Physical activity, sedentary behavior, and long-term cardiovascular risk in young people: a review and discussion of methodology in prospective studies. J Sport Health Sci.

[8] Skrede T, Steene-Johannessen J, Anderssen SA, Resaland GK, Ekelund U (2019). The prospective association between objectively measured sedentary time, moderate-to-vigorous physical activity and cardiometabolic risk factors in youth: a systematic review and meta-analysis. Obes Rev.

[9] van Ekris E, Altenburg TM, Singh AS, Proper KI, Heymans MW, Chinapaw MJM. An evidence-update on the prospective relationship between childhood sedentary behaviour and biomedical health indicators: a systematic review and meta-analysis. Obes Rev. 2016;17(9):833–49. 10.1111/obr.12426 27256486

[10] Berenson GS, Srinivasan SR, Nicklas TA. Atherosclerosis: a nutritional disease of childhood. Am J Cardiol. 1998;82(10B):22T–29T. 10.1016/s0002-9149(98)00719-x 9860370

[11] Flint J, Kothare SV, Zihlif M, Suarez E, Adams R, Legido A, De Luca F. Association between inadequate sleep and insulin resistance in obese children. J Pediatr. 2007;150(4):364–9. 10.1016/j.jpeds.2006.08.063 17382111

[12] Javaheri S, Storfer-Isser A, Rosen CL, Redline S. Sleep quality and elevated blood pressure in adolescents. Circulation. 2008;118(10):1034–40. 10.1161/CIRCULATIONAHA.108.766410 PMC2798149 18711015

[13] Javaheri S, Storfer-Isser A, Rosen CL, Redline S. Association of short and long sleep durations with insulin sensitivity in adolescents. J Pediatr. 2011;158(4):617–23. 10.1016/j.jpeds.2010.09.080 PMC3076647 21146189

[14] Klingenberg L, Chaput JP, Holmback U, Visby T, Jennum P, Nikolic M, Astrup A, Sjodin A. Acute sleep restriction reduces insulin sensitivity in adolescent boys. Sleep. 2013;36(7):1085–90. 10.5665/sleep.2816 PMC3669075 23814346

[15] Matthews KA, Dahl RE, Owens JF, Lee L, Hall M. Sleep duration and insulin resistance in healthy black and white adolescents. Sleep. 2012;35(10):1353–8. 10.5665/sleep.2112 PMC3443761 23024433

[16] Brooke HL, Atkin AJ, Corder K, Brage S, van Sluijs EM. Frequency and duration of physical activity bouts in school-aged children: A comparison within and between days. Prev Med Rep. 2016;4:585–90. 10.1016/j.pmedr.2016.10.007 PMC5107648 27843758

[17] Hjorth MF, Chaput JP, Michaelsen K, Astrup A, Tetens I, Sjodin A. Seasonal variation in objectively measured physical activity, sedentary time, cardio-respiratory fitness and sleep duration among 8–11 year-old Danish children: a repeated-measures study. BMC Public Health. 2013;13:808. 10.1186/1471-2458-13-808 PMC3846279 24010811

[18] Nordman M, Matthiessen J, Biltoft-Jensen A, Ritz C, Hjorth MF (2020). Weekly variation in diet and physical activity among 4-75-year-old Danes. Public Health Nutr.

[19] Rothausen BW, Matthiessen J, Hoppe C, Brockhoff PB, Andersen L, Tetens I. Differences in Danish children's diet quality on weekdays v. weekend days. Public Health Nutr. 2012;15(9):1653–60. 10.1017/S1368980012002674 22625874

[20] Hjorth MF, Damsgaard CT, Michaelsen KF, Astrup A, Sjodin A. Markers of metabolic health in children differ between weekdays - The result of unhealthier weekend behavior. Obesity. 2015;23(4):733–6. 10.1002/oby.21034 25755216

[21] Jaskolowski J, Ritz C, Sjodin A, Astrup A, Szecsi PB, Stender S, Hjorth MF. Weekday variation in triglyceride concentrations in 1.8 million blood samples. J Lipid Res. 2017;58(6):1204–13. 10.1194/jlr.M074062 PMC5454506 28381440

[22] Wedderkopp N, Jespersen E, Franz C, Klakk H, Heidemann M, Christiansen C, Moller NC, Leboeuf-Yde C. Study protocol. The Childhood Health, Activity, and Motor Performance School Study Denmark (The CHAMPS-study DK). BMC Pediatr. 2012;12:128. 10.1186/1471-2431-12-128 PMC3483192 22906115

[23] Friedewald WT, Levy RI, Fredrickson DS (1972). Estimation of the concentration of low-density lipoprotein cholesterol in plasma, without use of the preparative ultracentrifuge. Clin Chem.

[24] Marshall WA, Tanner JM. Variations in pattern of pubertal changes in girls. Arch Dis Child. 1969;44(235):291–303. 10.1136/adc.44.235.291 PMC2020314 5785179

[25] Marshall WA, Tanner JM. Variations in the pattern of pubertal changes in boys. Arch Dis Child. 1970;45(239):13–23. 10.1136/adc.45.239.13 PMC2020414 5440182

[26] Andersen LB, Andersen TE, Andersen E, Anderssen SA (2008). An intermittent running test to estimate maximal oxygen uptake: the Andersen test. J Sports Med Phys Fitness.

[27] Wamani H, Tylleskar T, Astrom AN, Tumwine JK, Peterson S. Mothers’ education but not fathers’ education, household assets or land ownership is the best predictor of child health inequalities in rural Uganda. Int J Equity Health. 2004;3(1):9. 10.1186/1475-9276-3-9 PMC529301 15482596

[28] Altenburg TM, Rotteveel J, Dunstan DW, Salmon J, Chinapaw MJ. The effect of interrupting prolonged sitting time with short, hourly, moderate-intensity cycling bouts on cardiometabolic risk factors in healthy, young adults. J Appl Physiol (1985). 2013;115(12):1751–6. 10.1152/japplphysiol.00662.2013 24136111

[29] An P, Borecki IB, Rankinen T, Despres JP, Leon AS, Skinner JS, Wilmore JH, Bouchard C, Rao DC. Evidence of major genes for plasma HDL, LDL cholesterol and triglyceride levels at baseline and in response to 20 weeks of endurance training: the HERITAGE Family Study. Int J Sports Med. 2005;26(6):414–9. 10.1055/s-2004-821160 16037881

[30] Taylor-Tolbert NS, Dengel D, Brown MD, McCole SD, Pratley RE, Ferrell RE, Hagberg JM. Ambulatory blood pressure after acute exercise in older men with essential hypertension. Am J Hypertens. 2000;13(1 Pt 1):44–51. 10.1016/s0895-7061(99)00141-7 10678270

[31] Dunstan DW, Kingwell B, Larsen R, Healy GN, Cerin E, Hamilton MT, Shaw JE, Bertovic DA, Zimmet PZ, Salmon J, Owen N. Breaking up prolonged sitting reduces postprandial glucose and insulin responses. Diabetes Care. 2012;35(5):976–83. 10.2337/dc11-1931 PMC3329818 22374636

[32] Peddie MC, Bone J, Rehrer NJ, Skeaff CM, Gray AR, Perry TL. Breaking prolonged sitting reduces postprandial glycemia in healthy, normal-weight adults: a randomized crossover trial. Am J Clin Nutr. 2013;98(2):358–66. 10.3945/ajcn.112.051763 23803893

[33] King DS, Baldus PJ, Sharp RL, Kesl LD, Feltmeyer TL, Riddle MS. Time course for exercise-induced alterations in insulin action and glucose tolerance in middle-aged people. J Appl Physiol (1985). 1995;78(1):17–22. 10.1152/jappl.1995.78.1.17 7713807

[34] Boule NG, Weisnagel SJ, Lakka TA, Tremblay A, Bergman RN, Rankinen T, Leon AS, Skinner JS, Wilmore JH, Rao DC, Bouchard C. Effects of exercise training on glucose homeostasis: the HERITAGE Family Study. Diab Care. 2005;28(1):108–14. 10.2337/diacare.28.1.108 15616242

[35] Cooper AR, Goodman A, Page AS, Sherar LB, Esliger DW, van Sluijs EM, Andersen LB, Anderssen S, Cardon G, Davey R (2015). Objectively measured physical activity and sedentary time in youth: the International children's accelerometry database (ICAD). Int J Behav Nutr Phys Act.

[36] Moller NC, Kristensen PL, Wedderkopp N, Andersen LB, Froberg K (2009). Objectively measured habitual physical activity in 1997/1998 vs 2003/2004 in Danish children: the European youth heart study. Scand J Med Sci Sports.

[37] Moller NC, Tarp J, Kamelarczyk EF, Brond JC, Klakk H, Wedderkopp N (2014). Do extra compulsory physical education lessons mean more physically active children--findings from the childhood health, activity, and motor performance school study Denmark (the CHAMPS-study DK). Int J Behav Nutr Phys Act.

[38] Kallio J, Hakonen H, Syvaoja H, Kulmala J, Kankaanpaa A, Ekelund U, Tammelin T (2020). Changes in physical activity and sedentary time during adolescence: gender differences during weekdays and weekend days. Scand J Med Sci Sports.

