# Supplementary material for: Weekly variation in markers of cardiometabolic health – the possible effect of weekend behavior – a cross-sectional study
Source: BMC Cardiovasc Disord. 2020 Sep 7;20:405. doi: 10.1186/s12872-020-01692-x (PMC7487626; doi:10.1186/s12872-020-01692-x)
Supplement: Supplementary file 1 — Additional file 1 Supplementary file 1. Self-assessment of pubertal status. Tool used in the CHAMPS study-DK III for judging participants’ sexual maturity [file 12872_2020_1692_MOESM1_ESM.pdf]

**Supplementary file 1.** Self-assessment of pubertal status

**Self-assessment of pubertal status (Girls)**

ID: _________ Date: __________

Age: _____ School: ____________________ Class: __________

**Pubic hair**

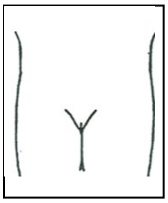

There is no pubic hair ❑

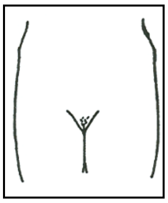

There is almost no, or very little, hair ❑

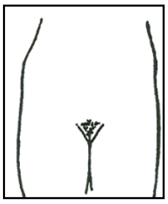

The pubic hair has become darker more dense ❑

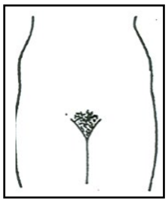

The pubic hair is almost like that of adult woman ❑

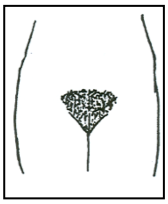

The pubic hair is like that of a young adult woman ❑

**Breast development**

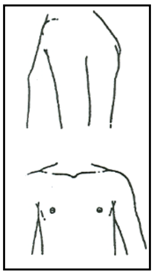

The nipple has not started growing ❑

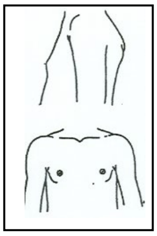

The nipple has just started growing pubic ❑

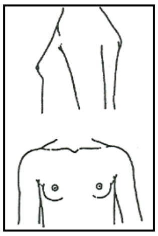

The breast itself has started growing and ❑

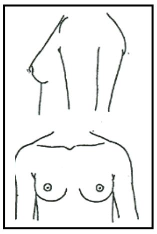

The breast is almost like that of a young young adult woman ❑

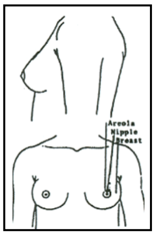


The breast has developed like that of young adult woman ❑

Menstruation debut: yes ❑ No ❑

**Self-assessment of pubertal status (Boys)**

ID: _________ Date: __________

Age: _____ School: ____________________ Class: __________

**Pubic hair**

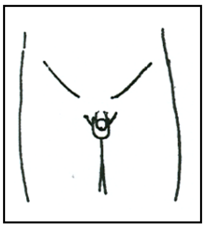

There is no pubic hair
❑

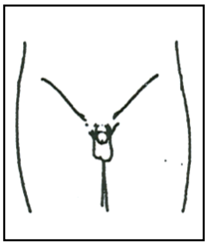

There is almost no, or very little, pubic hair
❑

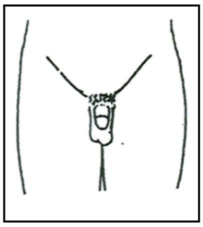

The pubic hair has become darker and more dense
❑

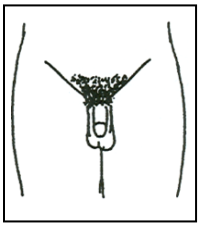

The pubic hair is almost like that of a young adult man
❑

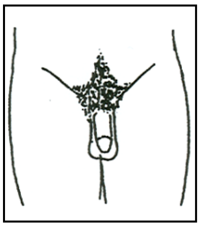

The pubic hair is like that of a young adult man
❑

(Note: In the original version, the drawings were accompanied by an explanatory text in Danish. Children were asked, separately and in privacy, to point out the drawing that they considered resembled their own pubertal stage the most).
