# Supplementary material for: Weekly variation in markers of cardiometabolic health – the possible effect of weekend behavior – a cross-sectional study
Source: BMC Cardiovasc Disord. 2020 Sep 7;20:405. doi: 10.1186/s12872-020-01692-x (PMC7487626; doi:10.1186/s12872-020-01692-x)
Supplement: Supplementary file 2 — Additional file 2 Supplementary file 2. Parents’ education and type of work. Questionnaire applied in the CHAMPS study-DK III for obtaining information on parental socioeconomic position [file 12872_2020_1692_MOESM2_ESM.pdf]

Supplementary file 2. Parents' education and type of work

1) **What is your highest education (please do not indicate any uncompleted education)?**

Mother/female guardian

1) ❑ Lower secondary school (9 years)
2) ❑ Lower secondary school (10 years)
3) ❑ Upper secondary school (e.g., gymnasium, Higher Commercial Education Programme, Higher Technical Education Programme)
4) ❑ Vocational education (e.g., business college (basic commercial course), internship training, Social and Health Care Training Programme, carpenter, bricklayer)
5) ❑ Short tertiary education (e.g., AP Graduate in Construction Technology, Commerce Management, or Computer Science, hygienist)
6) ❑ Medium tertiary education (e.g., bachelor or equivalent, schoolteacher, physiotherapist, nurse)
7) ❑ Long tertiary education (e.g., master or equivalent, graduate engineer, medical doctor)

Father/male guardian

1) ❑ Lower secondary school (9 years)
2) ❑ Lower secondary school (10 years)
3) ❑ Upper secondary school (e.g., gymnasium, Higher Commercial Education Programme, Higher Technical Education Programme)
4) ❑ Vocational education (e.g., business college (basic commercial course), internship training, Social and Health Care Training Programme), carpenter, bricklayer)
5) ❑ Short tertiary education (e.g., AP Graduate in Construction Technology, Commerce Management, or Computer Science, hygienist)
6) ❑ Medium tertiary education (e.g., bachelor or equivalent, schoolteacher, physiotherapist, nurse)
7) ❑ Long tertiary education (e.g., master or equivalent, graduate engineer, medical doctor)

2) What is your current employment / job position?

a) Mother/female guardian ______________________________

b) Father/male guardian ______________________________

3) How many hours a week do you work on average?

a) Mother/female guardian ______________________________

b) Father/male guardian ______________________________

4) Please fill in the form below if you do not have a job

	**Unemployed (unemployment insurance)**	**Social assistance recipients (welfare)**	**Voluntary early retiree**	**Early retirement**	**Retiree**	**Other**
a) Mother/female guardian	(1) ❑	(2) ❑	(3) ❑	(4) ❑	(5) ❑	(6) ❑ ____________
b) Father/male guardian	(1) ❑	(2) ❑	(3) ❑	(4) ❑	(5) ❑	(6) ❑ ____________
